# Nipah Virus Shedding in Urine from Fruit Bats, Sri Lanka, 2018–2019

**DOI:** 10.3201/eid3207.251567

**Published:** 2026-07

**Authors:** Claudia Kohl, Sahan Siriwardana, Therese Muzeniek, Thejanee Perera, Dilara Bas, Mizgin Öruc, Annika Brinkmann, Beate Becker-Ziaja, Franziska Schwarz, Hamsananthy Jeevatharan, Jagathpriya Weerasena, Shiroma Handunnetti, Inoka C. Perera, Gayani Premawansa, Sunil Premawansa, Wipula Yapa, Andreas Nitsche

**Affiliations:** Robert Koch Institute, Berlin, Germany (C. Kohl, T. Muzeniek, T. Perera, D. Bas, M. Öruc, A. Brinkmann, B. Becker-Ziaja. F. Schwarz, A. Nitsche); University of Colombo, Columbo, Sri Lanka (S. Siriwardana, T. Perera, J. Weerasena, S. Handunetti, I.C. Perera, S. Premawansa, W. Yapa); Ministry of Health, Colombo (H. Jeevatharan); Colombo North Teaching Hospital, Ragama, Sri Lanka (G. Premawansa)

**Keywords:** Nipah virus, Henipavirus, Pteropus giganteus, Pteropus medius, bats, viruses, henipa, zoonoses, Sri Lanka

## Abstract

Nipah virus causes outbreaks in humans with high case-fatality rates. In this study, we confirmed the presence of Nipah virus in Sri Lanka in *Pteropus medius* fruit bats, one of the known natural reservoir species. Sequences we generated were genetically related to Nipah virus strains from outbreaks in southern India.

Nipah virus (NiV; *Henipavirus nipahense*) and Hendra virus (*H. hendraense*) are species within the genus *Henipavirus* of the family Paramyxoviridae (*1*). Several paramyxoviruses, including measles virus and respiratory syncytial virus, cause serious respiratory illnesses and are often highly transmissible through the air. NiV can cause severe outbreaks in humans; its clinical symptoms range from subclinical infection to severe encephalitis, respiratory diseases, and death (*2*). Reported case-fatality rate (CFR) of NiV encephalitis is 61% (95% CI 45.7%–75.4%) (*2*,*3*). 

NiV emerged in 1998 in Malaysia and Singapore (*4*). Subsequent outbreaks were reported from Bangladesh, the Philippines, and India; they resulted in >643 laboratory-confirmed infections and 380 deaths (*2*). Bats (flying foxes, *Pteropus* spp.) are the known reservoir hosts of NiV (*5*). Transmission from bat to human occurs via exposure to urine, either through consumption of raw palm sap contaminated with bat excreta, direct contact with infected intermediate hosts (e.g., swine), or direct contact with bat urine (*5*). In 2023 several cases of Nipah virus were reported from Kozhikode, Kerala district, India, and in Dhaka, Rajbari, and Shariatpur districts in Bangladesh (*6*). The shortest distance between Mannar Island, Sri Lanka, and Natarajapuram, India, is <55 km; *Pteropus* spp. bats are reported to migrate >450 km with ease (*7*). In this study, we monitored urine excreted by several colonies of *P. medius* bats in Sri Lanka for the presence of viruses using molecular techniques. Our focus was to investigate if NiV is present in Sri Lanka and to determine the measures required to prevent spillover into humans. We obtained ethics approval for this study from the Institute of Biology, Sri Lanka (WL/3/2/05/18) and necessary clearance from the Department of Wildlife Conservation, Sri Lanka.

## The Study

During March 2018–June 2019, we monitored *P. medius* bat colonies for shedding of pathogens in Colombo, Mannar, Anuradhapura, and Badulla, Sri Lanka. We laid clean sampling sheets below roosting trees in the early morning and transferred excreted urine drop by drop to sterile microtiter plates in the morning using disposable pipettes. We conducted all work while wearing appropriate personal protective equipment (PPE) as previously described (*5*).

We collected a total of 2,218 urine samples and combined them into 32 pools with <8 urine specimens each. We extracted RNA from the individual pools using the QIAamp Viral RNA Mini Kit (QIAGEN, https://www.qiagen.com). We performed Nipah virus screening using real-time PCR targeting the phosphoprotein (P) gene, as previously described (*8*). As an additional confirmation, we developed a real-time PCR targeting the nucleocapsid protein of the Nipah virus genome (Table 1).

**Table 1:** Primers and cycling conditions for quantitative PCR assay developed for study of NiV shedding in urine from fruit bats, Sri Lanka, 2018–2019

Component	Conditions
Primers	
NiV_F	AAA TCA AGT TGC AGA ACT CGC T
NiV_R	CTC CRA TGA GCA CAC CTC CTG
NiV Probe	FAM-CTT CCT GCT GAT GTT TC- MGB
Protocol†	AgPath-ID One-Step RT-PCR Kit,‡ 6 µL H_2_0, 12.5 µL RT buffer, 1 µL NiV_F (10 µM), 1 µL NiV_R (10 µM), 0.5 µL NiVN_probe (10 µM), 1.0 µL enzyme mix (25×)
Cycling conditions	45°C for 900 s, 95°C for 600 s, 95°C for 15 s for 45 cycles, 60°C for 45 s
Cycler types	Light Cycler 480,§ ABI 7500,‡ Bio-Rad CFX 96¶

*F, forward primer; FAM, fluorescein amidites; MGB, minor groove binder; NiV, Nipah virus; R, reverse primer. †The primers listed are all included in the mix. ‡Applied Biosystems, https://www.thermofisher.com. §Roche Life Science, https://lifescience.roche.com. ¶Bio-Rad Laboratories, https://www.bio-rad.com.

**Figure 1:**
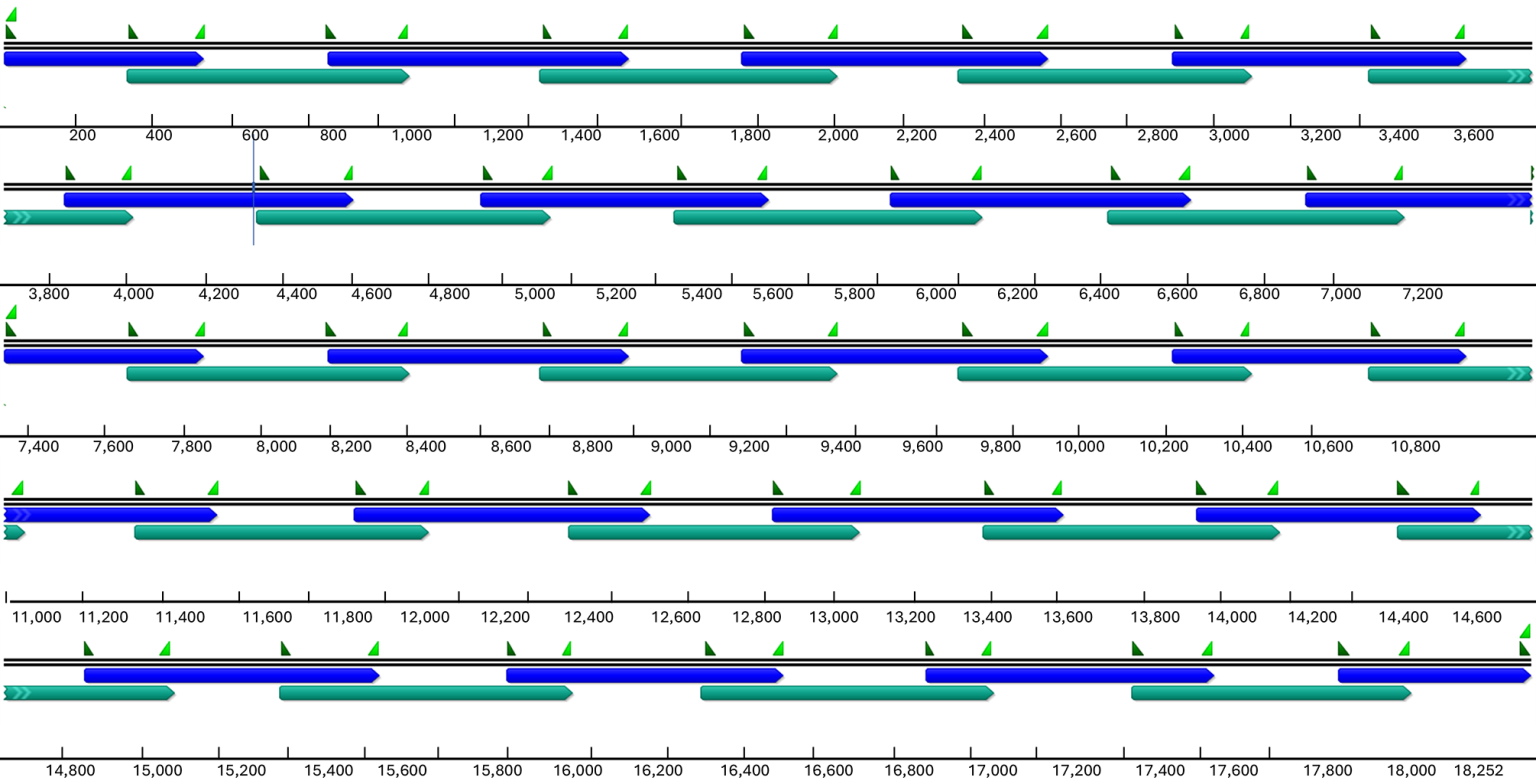
Positions of primers used for NiVliSeq amplification panel in study of Nipah virus shedding in urine from fruit bats, Sri Lanka, 2018–2019. Blue and green represent 2 nonoverlapping primer pools to which the primers were added.

We used Illumina HiSeq (https://www.illumina.com) to sequence the NiV positive pools S3_P02 and S18_P08 (2 of 32 pools). We trimmed using Trimmomatic (*9*) and mapped to NiV strain MCL-18-H-1088 (accession no. MHK523642; Kerala, India) as a reference strain. In addition, we developed an AmpliSeq primer panel, NiVliSeq, spanning the whole NiV genome using Primer3 version 2.3.7 (Figure 1; Appendix Table). We sequenced corresponding PCR products on the MinIon platform as described previously (*10**,**11*). We submitted draft genomes to GenBank (accession nos. PP893186–9) and aligned them with NiV strains and type species of paramyxovirus genomes indicated by the International Committee on Taxonomy of Viruses and available in GenBank. We calculated phylogenetic molecular clock reconstruction with MrBayes version 0.39 software (https://nbisweden.github.io/MrBayes) using Markov chain Monte Carlo approach of molecular clock reconstruction based on the general time reversible model with the parameters set at 1 million replicates, 4 chains, and burn in at 10% (partial large sequence 7,977 nt; coding for the RNA-dependent RNA polymerase gene, of the subfamily Orthoparamyxovirinae).

We collected pool S3_P02 and pool S18_P08 in Colombo; both tested positive for NiV using the henipavirus quantitative PCR (qPCR) targeting the P gene (*8*). The NiV qPCR (targeting the nucleocapsid gene) further confirmed NiV in the same sample pools (Table 2). Sequencing revealed corresponding NiV reads in the 2 sample pools that we mapped over the entire NiV genome (Table 2), providing additional confirmation. The NiVliSeq approach revealed 93% of the NiV genome from pool S3_P02 and 98% of NiV genome for pool S18-08, (Table 2). Comparison of the NiV genomes with the GenBank database revealed the highest identity to a strain collected from a human patient during the 2018 outbreak in Kerala, India (accession no. MH396625); the genomes from pool S3_P02 and pool S18-08 each had 98% nucleotide identity to the Kerala strain. We named the Sri Lanka NiV strains in accordance with the system used for the India strains: NiV strain C-18-B-0302 Sri Lanka (accession no. PP893186.1) and NiV strain C-19-B-1808 Sri Lanka (accession no. PP893188.1). In that nomenclature, C denotes Colombo, 18 and 19 indicate the years 2018 and 2019, B denotes bat, and the remaining numbers represent the sample number.

**Table 2:** Sequencing results for NiV-positive bat urine samples in study of NiV shedding in urine from fruit bats, Sri Lanka, 2018–2019*

Characteristic	Bat urine pools tested positive for NiV	
	S03-P2	S18-08
Origin	*Pteropus medius*, Colombo	*P. medius*, Colombo
Sampling date	June 2018	March 2019
PCR		
Henipa PCR		
Ct value	33	35
Mean duplicates	8	6
NiV in-house		
Ct value	32	37
Mean duplicates	3	7
Illumina sequencing, shotgun†		
Trimmed reads	5,094,516	5,633,085
NiV reads	128	20
Longest contig	481 nt (accession no. PP893187.1)	255 nt (accession no. PP893189.1)
Highest % ID nt	98.96% MCL-18-H-1088 (accession no. MHK523642)‡	99.61% (accession no. MN549404.1)§
NiVliSeq, amplicon-based approach¶		
Genome length and coverage	93% query, 189-fold average (>10-fold)	98% query, 8,783-fold average (>10-fold)
Highest % ID nt	98.34%‡	98.43%‡
Novel strain	Nipah virus strain C-18-B-0302 Sri Lanka (accession no. PP893186.1)	Nipah virus strain C-19-B-1808 Sri Lanka (accession no. PP893188.1)

*Ct, cycle threshold; NiV, Nipah virus; % ID, percentage identity of nucleotide and amino acid sequence between pool and reference strain. †Illumina, https://www.illumina.com. ‡Reference strain MH396625 (GenBank accession no.) from a human patient during the 2018 outbreak in Kerala, India. §Reference strain from a bat in India. ¶ AmpliSeq primer panel spanning the whole NiV genome using Primer3 version 2.3.7 (Figure 1; Appendix Table).

**Figure 2:**
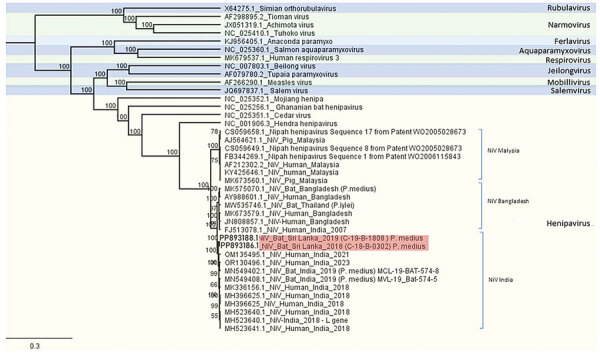
Phylogenetic reconstruction of subfamily Orthoparamyxovirinae viruses in study of NiV shedding in urine from fruit bats, Sri Lanka, 2018–2019. The alignment is based on the partial large gene (7,977 nt) coding for the RNA-dependent RNA polymerase. Calculation was done using a MrBayes Markov chain Monte Carlo approach of molecular clock reconstruction based on the general time reversible model with the parameters set at 1 million replicates, 4 chains, and burn-in at 10%. Blue shading and green shading indicate distinct genera of paramyxoviruses. Red shading highlights the novel strains from Sri Lanka. Node numbers indicate posterior probability. We used a simian orthorubulavirus as an outgroup. Scale bar indicates branch length. NiV, Nipah virus; *P. medius*, *Pteropus medius*.

Phylogenetic analysis allocated the novel Nipah virus strains C-18-B-0302 Sri Lanka and C-19-B-1808 Sri Lanka to the species Nipah virus (*H. nipahense*) within the genus *Henipavirus* (Figure 2). Both are clustering monophyletically with the strains sampled from humans during the outbreaks in Kerala region, India in 2007, 2018, 2019, 2021, and 2023.

## Conclusions

This study demonstrated the presence of NiV strains in Sri Lanka. The qPCR and NiVliSeq sequencing approach we developed validated previously obtained results and represented valuable tools that can be used in further studies. The identified strains have highest similarities to human-pathogenic strains causing recent outbreaks in India (2007, 2018, 2019, and 2023). However, NiV is still a rare disease, and transmission rates are comparably low. Furthermore, the detection of a certain virus in a bat population does not necessarily mean that transmission occurs; diverse strains of henipaviruses can be found in *Pteropus* spp. bats across the whole distribution range. 

The initial prevention of any infection should be one of the major goals and could include protection of the bats and their roosts. Bat colonies are vital for healthy ecosystems and should be protected for several reasons, including the ecosystem services they provide such as pollination and seed dispersal. Moreover, *Pteropus* spp. bats are threatened by extinction (*12*). The World Health Organization (WHO) has recognized the need for research and diagnosis of NiV within the distribution range of *Pteropus* spp. (*13*).

Understanding the mechanisms influencing shedding of NiV will likely prevent further spread; NiV shedding likely also follows seasonal and temporal pulses previously reported for Hendra virus (*14*–*15*). We recognize that disease emergence might be a result of disturbance within ecologic systems and hence cannot be alleviated by further disturbance. As a tropical country, Sri Lanka has complex and diverse ecosystems; its resilience to climate change may be better than other regions of the world. 

The potential of zoonotic bat-to-human transmission of NiV could be minimized by preventive measures. Such measures could include education of healthcare workers to raise appropriate awareness for NiV, equipment and training for the early detection of NiV RNA in clinical specimens, preparation for clinical management of NiV-positive patients, and messaging the public about avoidance of known infection routes to prevent transmission (i.e., circular fencing of trees with roosting bats).

Appendix Additional information on primers used in study of Nipah virus shedding in urine from fruit bats, Sri Lanka, 2018–2019.
